# Complete genome sequence of *Thioalkalivibrio paradoxus* type strain ARh 1^T^, an obligately chemolithoautotrophic haloalkaliphilic sulfur-oxidizing bacterium isolated from a Kenyan soda lake

**DOI:** 10.1186/s40793-015-0097-7

**Published:** 2015-11-19

**Authors:** Tom Berben, Dimitry Y. Sorokin, Natalia Ivanova, Amrita Pati, Nikos Kyrpides, Lynne A. Goodwin, Tanja Woyke, Gerard Muyzer

**Affiliations:** Microbial Systems Ecology, Department of Aquatic Microbiology, Institute for Biodiversity and Ecosystem Dynamics, University of Amsterdam, Amsterdam, The Netherlands; Winogradsky Institute of Microbiology, RAS, Moscow, Russia; Department of Biotechnology, Delft University of Technology, Delft, The Netherlands; Joint Genome Institute, Walnut Creek, California, USA; Department of Biological Sciences, Faculty of Science, King Abdulaziz University, Jeddah, Saudi Arabia

**Keywords:** Haloalkaliphilic, Soda lakes, Sulfur-oxidizing bacteria, Thiocyanate

## Abstract

*Thioalkalivibrio paradoxus* strain ARh 1^T^ is a chemolithoautotrophic, non-motile, Gram-negative bacterium belonging to the *Gammaproteobacteria* that was isolated from samples of haloalkaline soda lakes. It derives energy from the oxidation of reduced sulfur compounds and is notable for its ability to grow on thiocyanate as its sole source of electrons, sulfur and nitrogen. The full genome consists of 3,756,729 bp and comprises 3,500 protein-coding and 57 RNA-coding genes. This organism was sequenced as part of the community science program at the DOE Joint Genome Institute.

## Introduction

Soda lakes are characterized by a high and stable pH (>9) due to the presence of molar concentrations of soluble carbonates as the dominant anions and a moderate to high salinity [1]. They are found in arid zones in many parts of the world, for example, in the Kulunda Steppe in Russia, North-Eastern China, the Rift Valley in Africa and the arid regions of California and Nevada (e.g., Mono Lake, Big Soda Lake). Despite their (extremely) haloalkaline character, these environments harbor a rich microbial diversity that is responsible for driving highly active biogeochemical cycles [2], of which the sulfur cycle is the most active. Our current research focuses on a group of chemolithoautotrophic sulfur-oxidizing bacteria that belong to the genus *Thioalkalivibrio* in the class *Gammaproteobacteria*. These organisms are of interest because of their role in the oxidative part of the sulfur cycle in soda lakes [3] and their application in the sustainable removal of sulfur from wastewater and gas streams [4]. To better understand the success of this group of organisms, we have sequenced the genomes of a large number of *Thioalkalivibrio* isolates. Here we present the genome sequence of *T. paradoxus* ARh 1^T^ (= DSM 13531^T^ = JCM 11367^T^).

## Organism information

### Classification and features

**Fig. 1:**
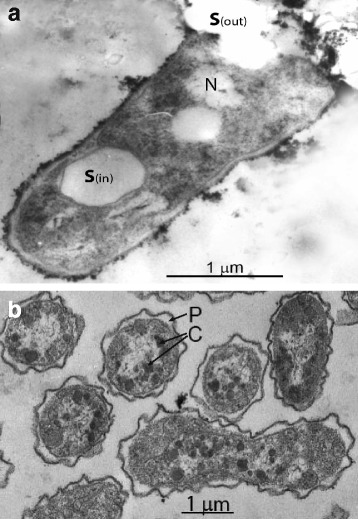
Thin section electron microscopy photographs of cells of strain ARh 1^T^ grown with thiocyanate in batch (**a**) and chemostat (**b**) cultures at pH 10 and 0.6 M total Na^+^. **S**(in) - intracellular sulfur globe; **S**(out) - excreted sulfur globe damaging the cell membrane and the wall; **N** - nucleoid; **C** - carboxysomes

**Fig. 2:**
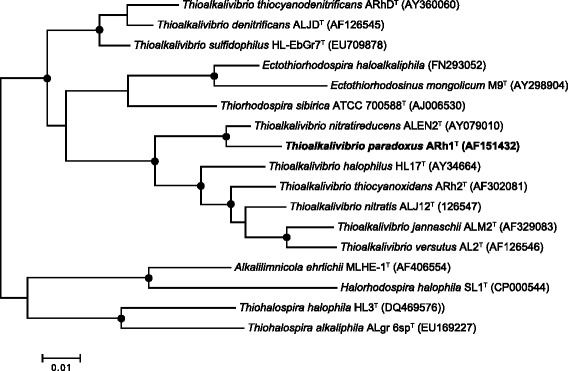
Phylogenetic tree, based on 16S rRNA sequences, of *Thioalkalivibrio* and various members of the *Ectothiorhodospiraceae* family. ARB [22] was used for tree construction and MEGA6 [23] for the bootstrap analysis. *Alphaproteobacteria* were used as the outgroup and pruned from the finished tree

This obligate aerobic and haloalkaliphilic strain, which was isolated from a mixed sample of sediments from Kenyan soda lakes, is a non-motile coccoid rod forming intracellular sulfur as an obligate intermediate during oxidation of thiosulfate and thiocyanate (Fig. 1). It is an obligate chemolithoautotroph, capable of using a variety of reduced, inorganic sulfur compounds, including sulfide, thiosulfate and polysulfide, as electron donor for carbon fixation. It can also oxidize CS_2_ (carbon disulfide). Of special interest is its ability to grow with thiocyanate (NCS^−^) as electron donor, with a relatively high growth rate of 0.08–0.1 h^−1^ in continuous culture, compared to 0.01–0.015 h^-^^1^ for growth on thiosulfate [5]. Phylogenetic analysis based on 16S rRNA sequences shows that *T. paradoxus* is closely related to *Thioalkalivibrio nitratireducens* ALEN 2^T^ (Fig. 2). An overview of basic features of the organism is provided in Table 1.

**Table 1:** Classification and general features of *Thioalkalivibrio paradoxus ARh 1* ^T^ [24]

MIGS ID	Property	Term	Evidence code^a^
	Classification	Domain *Bacteria*	TAS [25]
		Phylum *Proteobacteria*	TAS [26, 27]
		Class *Gammaproteobacteria*	TAS [27, 28]
		Order *Chromatiales*	TAS [27, 29]
		Family *Ectothiorhodospiraceae*	TAS [30]
		Genus *Thioalkalivibrio*	TAS [31]
		Species *Thioalkalivibrio paradoxus*	TAS [5]
		Type strain: ARh 1^T^ (DSM 13531)	
	Gram stain	Negative	TAS [5, 31]
	Cell shape	Barrel-like rods	TAS [5]
	Motility	Non-motile	TAS [5]
	Sporulation	Non-sporulating	NAS
	Temperature range	Mesophilic	TAS [5]
	Optimum temperature	35–37 °C	TAS [5]
	pH range; Optimum	8.5–10.5	TAS [5]
	Carbon source	Inorganic carbon	TAS [5]
MIGS-6	Habitat	Soda lakes	TAS [5]
MIGS-6.3	Salinity	0.3–1.0 M Na^+^	TAS [5]
MIGS-22	Oxygen requirement	Aerobe	TAS [5]
MIGS-15	Biotic relationship	free-living	NAS
MIGS-14	Pathogenicity	Non-pathogenic	NAS
MIGS-4	Geographic location	Kenya	TAS [5]
MIGS-5	Sample collection	1999	TAS [5]
MIGS-4.1	Latitude	Not reported	
MIGS-4.2	Longitude	Not reported	
MIGS-4.4	Altitude	Not reported	

^a^Evidence codes - IDA: Inferred from Direct Assay; TAS: Traceable Author Statement (i.e., a direct report exists in the literature); NAS: Non-traceable Author Statement (i.e., not directly observed for the living, isolated sample, but based on a generally accepted property for the species, or anecdotal evidence). These evidence codes are from the Gene Ontology project [32]

## Genome sequencing information

### Genome project history

In order to better understand the diversity within the genus *Thioalkalivibrio*, as well as their biogeochemical role in soda lakes, a large number of isolates (approximately 70) was sequenced at the Joint Genome Institute. The full genome of the type strain of *Thioalkalivibrio paradoxus* presented here contains 3.8 million basepairs. Sequencing was performed at the JGI under project number 401912 and the sequence data was subsequently released in Genbank on December 31, 2013. A project overview is provided in Table 2.

**Table 2:** Project information

MIGS ID	Property	Term
MIGS 31	Finishing quality	Finished
MIGS-28	Libraries used	Illumina
MIGS 29	Sequencing platforms	Illumina HiSeq 2000
MIGS 31.2	Fold coverage	1,486X
MIGS 30	Assemblers	Velvet [8], ALLPATHS R39750 [7]
MIGS 32	Gene calling method	Prodigal [14], GenePRIMP [15]
	Locus Tag	THITH
	Genbank ID	NZ_CP007029
	GenBank Date of Release	2013–12–31
	GOLD ID	Gp0008932
	BIOPROJECT	PRJNA52643
MIGS 13	Source Material Identifier	DSM 13531
	Project relevance	Biotechnology

**Table 3:** Genome statistics

Attribute	Value	% of Total
Genome size (bp)	3,756,729	100
DNA coding (bp)	3,305,445	87.99
DNA G + C (bp)	2,500,004	66.55
Total genes	3,557	100
Protein coding genes	3,500	98.40
RNA genes	57	1.60
Pseudo genes	124	3.49
Genes in internal clusters	176	3.46
Genes with function prediction	2,739	77.00
Genes assigned to COGs	2,317	65.14
Genes with Pfam domains	2,835	79.70
Genes with signal peptides	271	7.62
Genes with transmembrane helices	841	23.64
CRISPR repeats	8	100

### Growth conditions and genomic DNA preparation

A buffer using sodium carbonate and bicarbonate, with a total salt concentration of 0.6 M Na^+^, was used for cultivation of the organism; the energy source was thiosulfate (40 mM). After harvesting, the cells were stored at −80 °C for further processing. Genomic DNA was extracted using a standard chloroform-phenol-isoamyl alcohol mixture, followed by ethanol precipitation. After vacuum drying, the pellet was dissolved in water and the quantity and quality of the DNA determined using the JGI-provided Mass Standard Kit.

### Genome sequencing and assembly

The draft genome of *Thioalkalivibrio paradoxus* ARh 1^T^ was generated at the DOE Joint Genome Institute (JGI) using Illumina data [6]. For this genome, we constructed and sequenced an Illumina short-insert paired-end library with an average insert size of 270 bp which generated 18,589,770 reads and an Illumina long-insert paired-end library with an average insert size of 7,058.67 +/–3247.54 bp which generated 20,051,794 reads totaling 5,796 Mbp of Illumina data (unpublished, Feng Chen). All general aspects of library construction and sequencing performed at the JGI can be found at http://www.jgi.doe.gov/. The initial draft assembly contained 83 contigs in 11 scaffolds. The initial draft data was assembled with ALLPATHS [7], version 39750, and the consensus was computationally shredded into 10 Kbp overlapping fake reads (shreds). The Illumina draft data was also assembled with Velvet, version 1.1.05 [8], and the consensus sequences were computationally shredded into 1.5 Kbp overlapping fake reads (shreds). The Illumina draft data was assembled again with Velvet using the shreds from the first Velvet assembly to guide the next assembly. The consensus from the second Velvet assembly was shredded into 1.5 Kbp overlapping fake reads. The fake reads from the ALLPATHS assembly and both Velvet assemblies and a subset of the Illumina CLIP paired-end reads were assembled using parallel phrap, version 4.24 (High Performance Software, LLC). Possible mis-assemblies were corrected with manual editing in Consed [9–11]. Gap closure was accomplished using repeat resolution software (Wei Gu, unpublished), and sequencing of bridging PCR fragments with Sanger and/or PacBio (unpublished, Cliff Han) technologies. A total of 50 additional sequencing reactions were completed to close gaps and to raise the quality of the final sequence. The size of the genome is 3.8 Mb and the final assembly is based on 5,796 Mbp of Illumina draft data, which provides an average 1,486X coverage of the genome.

### Genome annotation

The assembled sequence was annotated using the JGI prokaryotic annotation pipeline [12] and was further reviewed using the Integrated Microbial Genomes - Expert Review (IMG-ER) platform [13]. Genes were identified using Prodigal [14], followed by manual curation using GenePRIMP [15]. Predicted CDSs were translated and used to search the NCBI non-redundant, UniProt, TIGRFam, Pfam, KEGG, COG and InterPro databases. The tRNAScanSE tool [16] was used to detect tRNA genes and ribosomal RNA genes were detected using models contructed from SILVA [17]. Other RNA genes were predicted using Rfam profiles in Infernal [18]. CRISPR elements were detected using CRT [19] and PILER-CR [20]. Further annotation was performed using the Integrated Microbial Genomics (IMG) platform [21].

## Genome properties

**Fig. 3:**
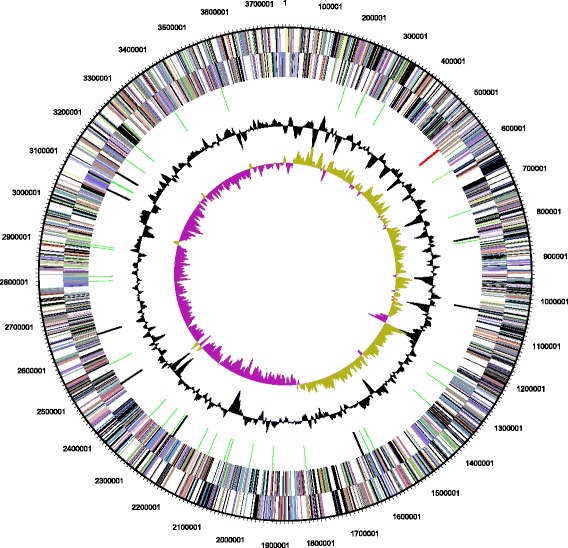
Genome map of *Thioalkalivibrio paradoxus* ARh 1^T^. From outer to inner ring: genes on the forward strand; genes on the reverse strand; RNA genes (tRNA: green; rRNA: red; other: black); GC content and GC skew

The finished genome with a G + C percentage of 66.06 % comprises a single chromosome of approximately 3.8 Mb (Fig. 3). There are 3557 genes of which 3,500 are protein-coding genes (a summary of genome properties is shown in Table 3). Approximately two-thirds of the protein coding genes could be assigned to a COG functional category (Table 4).

**Table 4:** Number of genes associated with the 25 general COG functional categories

Code	Value	Percent	Description
J	204	7.95	Translation, ribosomal structure and biogenesis
A	2	0.08	RNA processing and modification
K	103	4.02	Transcription
L	96	3.74	Replication, recombination and repair
B	1	0.04	Chromatin structure and dynamics
D	32	1.25	Cell cycle control, Cell division, chromosome partitioning
V	116	4.52	Defense mechanisms
T	119	4.64	Signal transduction mechanisms
M	201	7.84	Cell wall/membrane biogenesis
N	34	1.33	Cell motility
U	51	1.99	Intracellular trafficking and secretion
O	158	6.16	Posttranslational modification, protein turnover, chaperones
C	228	8.89	Energy production and conversion
G	91	3.55	Carbohydrate transport and metabolism
E	162	6.32	Amino acid transport and metabolism
F	61	2.38	Nucleotide transport and metabolism
H	150	5.85	Coenzyme transport and metabolism
I	95	3.70	Lipid transport and metabolism
P	178	6.94	Inorganic ion transport and metabolism
Q	36	1.40	Secondary metabolites biosynthesis, transport and catabolism
R	237	9.24	General function prediction only
S	146	5.69	Function unknown
-	1,240	34.86	Not in COGs

The total is based on the total number of protein coding genes in the genome

## Conclusions

The availability of high-quality genomic sequences of the type strains of *Thioalkalivibrio*, the dominant genus of sulfur-oxidizing bacteria in soda lakes, is an invaluable tool for gaining a more complete understanding of the biogeochemistry of these extreme environments. Additionally, this information may provide new insights into the exact mechanisms of adaptation these bacteria have evolved to not only survive, but thrive in this habitat. Finally, the genome may contain clues that will help improve the existing biotechnological applications of this organism in bioremediation.
